# Expanded and unclear responsibilities: the evolving role of home care workers as a lifeline during the COVID-19 pandemic -a focus group interview study

**DOI:** 10.1186/s12913-025-13145-2

**Published:** 2025-08-22

**Authors:** Karin Bölenius, Fredrik Norström, Malin Öhrling, Klas-Göran Sahlen, Anita Pettersson-Strömbäck

**Affiliations:** 1 Department of Nursing, Umeå, Sweden; 2 Department of Epidemiology and Global Health, Umeå, Sweden; 3 Department of Psychology, Umeå, Sweden

**Keywords:** COVID-19, Experiences, Health, Home care services, Staff, Work environment

## Abstract

**Background:**

Home care services, with the aim to support older adults in their homes, faced intense external pressure to create sustainable working conditions for staff during the COVID-19 pandemic. Studies have indicated elevated burnout and stress among residential care staff, and it is likely that similar challenges exist in home care services. Overall, the consequences for staff's work environment and health under the extremely strained conditions of the COVID-19 pandemic merit closer examination. Therefore, the aim is to illuminate home care workers’ experiences of their work environment and health risks while supporting older adults during the COVID-19 pandemic.

**Methods:**

To gain an in-depth understanding of personal experiences during the pandemic, five focus group interviews (FG) were held in northern Sweden during spring 2022. Open-ended questions were delivered via a semi-structured interview guide. Thematic analysis was used to guide data analysis.

**Results:**

Expanded and unclear responsibilities characterized the work environment during the pandemic. This was summarized into four sub-themes: my own health was jeopardized; a wind of change towards more responsibility; struggling between being a lifeline and being contagious; and organization and management as facilitators or hindrances.

**Conclusions:**

The findings underscore the importance of management strategies and organizational preparedness to support staff’s mental health and mitigate challenges during crises. The health risks associated with increased workload, stress, and mental burdens were evident in the narratives. The study emphasizes the need to strive for a good work environment, set priorities to reduce workloads and mental stress, and incorporate time for recovery among staff during crises. It is also of great importance that different authorities in healthcare and social care create effective cooperation so that information, knowledge, and policies are effectively disseminated to frontline staff who have the concrete responsibility for our elderly.

**Supplementary Information:**

The online version contains supplementary material available at 10.1186/s12913-025-13145-2.

## What is already known about this topic

- Elevated trauma, burnout, and stress reported by residential care staff due to suspended family involvement during Covid-19
- Job responsibilities and work practices have changed due to an increasing number of older adults
- Pre-Covid studies showed 28% of homecare workers faced high workloads with unevenly distributed tasks
- High workloads are linked to health problems for workers

## What this paper adds

- Examines how various and changing tasks in homecare services impact staff's work environment and health risks
- Highlights that work tasks have evolved, with new tasks added and some previous tasks re-prioritized or deprioritized
- Emphasizes the need for further study on the consequences of the extremely strained working conditions during crises on staff's environment and health

## Background

Home care services enable older adults to age in place—i.e., at home—for as long as they desire. Both globally and also in Sweden, we are facing a demographic transition with an increase in the proportion of older adults in the population and in life expectancy [1–3], commonly referred as “the silver tsunami”. In recent years, this has meant that more healthy older adults continue to stay in their homes for an extended period, as do older adults with increased care needs. These increased care needs place greater demands on staff, most of whom have a high school education and limited care experience. Additionally, job responsibilities and work practices have changed to meet the growing number of older adults and their increased care needs, both in Sweden and internationally [2–4]. In autumn 2019, the media began reporting on an unknown virus infection identified as COVID-19. By 2020, WHO classified the COVID-19 virus as a pandemic [5]. The pandemic has resulted in severe effects on mortality and morbidity as well as effects on the global economy, education, and the work environment [6]. There is a knowledge gap regarding the experiences of home care services staff concerning their work environment and health during the COVID-19 pandemic, as research has primarily focused on residential care.

Research shows that older adults residing in residential care facilities are particularly vulnerable to viral infections [7]. To promote healthy aging, a robust workforce and a favorable work environment for staff are essential [8]. In connection with the latter, research suggests that pre-existing safety and health climates may act as a protective factor for staff well-being, even in the face of crises such as COVID-19 [9]. Studies indicate that staff in residential care facilities reported elevated levels of trauma, burnout, and stress, with adverse consequences stemming from the suspension of family involvement during COVID-19 [10]. Triggers for negative psychosocial effects related to work include fear of infection and spreading the virus, lack of recognition from employers, absence of guidance, unsafe hospital discharge, death and the subsequent loss of professionals and residents, unreliable testing with delayed results, and staff shortages [11]. It is reasonable to believe that the same applies to home care services. One study from the United States on home care services in relation to COVID-19 shows that staff perceived that the pandemic implied challenges that aggravated their feelings of being a marginalized work force [12].

One study conducted in Sweden before the COVID-19 pandemic reported that 28% of participants identified as having a high workload in home care service faced challenges related to unevenly distributed work and an excess of working tasks. Issues that lead to high workload outweighed those related to learning demands, and workload-related challenges were mostly observed across groups linked to health education, i.e. to either have formal training as a nurse assistant or to have another type of education [13]. Research has also shown that high workloads are linked to health problems for workers in the healthcare sector and in the care of older adults such as chronic fatigue syndrome, burn out, ethical stress, and a worsened quality of life [13–17] and increased rates of illness [17–19] even before COVID-19. The job demand-resource model (JD-R) explains work-related stress as an imbalance between perceived job demands and the perceived resources that workers have as a buffer to these demands. Both job demands and resources originates steams from physical, psychological, social, and/or organizational aspects of the work [20, 21]. Aspects of work that are experienced, such as either a demand or a resource are context specific, i.e., they differ across work tasks and organizations. The JD-R model states that there are two processes going on at work: a health impairment process, in which high job demands yield strain and health debilitation,and a motivational process, in which access to job resources enhances motivation and performance and buffers against work demands. It is reasonable to believe that home care workers experience an imbalance between job demands and perceived resources during crisis.

In Sweden, the responsibility for elder care is shared between municipalities and regions. Municipalities are responsible for helping and supporting individuals in daily life. Regions are primarily responsible for medical care for older adults, but in some cases this responsibility is shared with the municipalities. Decades of increased efficiency requirements, restructuring and new management principles inspired by New Public Management (NPM), have had a great impact [22–24]. The challenges that home care services have faced during the pandemic have required adaptation, flexibility, and cooperation, as well as good leadership. How leadership in elderly care is exercised has been shown to be of great importance [25, 26]. Whether these prerequisites can be met by an organizational logic characterized by standards, routines, measurable outcomes, evaluations, and a hierarchical division of labor is an empirical question.

In short, during COVID-19, home care has been exposed to strong external pressure not only to protect users but also to create sustainable working conditions for staff. Ambitions regarding a minimized risk of infection have been expressed while shortcomings in home care service have been pointed out (e.g., a lack of protective equipment, high proportion of hourly staff, and staff without sufficient training) by the media. Further, the COVID-19 pandemic can be assumed to have meant that work tasks have changed, that new ones have been added, and that certain previous tasks have been re-or de-prioritized. At present, knowledge is rather scarce about home care workers experiences of how the various and changing tasks within home care services during the COVID-19 pandemic affected their work environment and health. Thus, there is a need of research, and more specifically, research with a qualitative, inductive, meaning-making approach, to create a deeper understanding and knowledge about this contemporary and unknown social phenomenon [27, 28]. Overall, it can be stated that the consequences for the staff's work environment and health under the extremely strained conditions that applied during the COVID-19 pandemic need to be studied more closely.

### Aim

The overarching aim of the study was to illuminate home care workers experiences of their work environment and health risks while supporting older adults during the COVID-19 pandemic with the following research questions:

- 1) How did the staff experience that their work environment was affected during the Covid pandemic?
- 2) Did the staff experiences of perceived changes in the work environment impact their health?
- 3) What challenges and solutions did the staff identify in response to the perceived new demands on operations due to the COVID-19 pandemic?

## Methods

### Design

To gain an in-depth understanding of personal experiences and perceptions during the pandemic, focus group interviews (FG) were conducted during spring 2022. FGs are a suitable data collection method when you want to facilitate communication and interaction among participants who share the same interest and cultural background but have different point of views, with the aim of collecting high-quality data in a short amount of time [29].

The present study was conducted as part of a larger project, Work Environment and Working Conditions (WeWorC). WeWork started in 2017 among homecare staff in three counties in Northern Sweden with the aim of exploring the work environment within home care service [13]. For this study, the overall project, with an established network of possible respondents, provided a great opportunity to select staff with different points of view and a variation in gender, age, and length of employment. The selection of participants was done strategically in 2022 by contacting respondents from the larger project who had indicated their willingness to participate in future interviews. Based on these criteria, we assembled the focus group to ensure a spread in the variables.

### Context/Setting

The responsibility for older adults who need help is shared between municipalities and regions in Sweden. Municipalities are responsible for providing help and support in daily life, while regions are primarily responsible for medical care. In some instances, such as home care, the responsibility for medical and nursing care lies with the county councils´ healthcare, while administration of healthcare is shared between the county council's healthcare and with municipalities. During 2022, home care in Sweden was characterized by recruitment difficulties [30].

In Sweden, relatively few and limited COVID-19 measures were used compared to many other countries. No general lockdown was imposed, and face masks were only recommended during a limited period and only in public transportation. Gatherings of more than 500 people were prohibited, and the public was urged to maintain distance and avoid large social gatherings. during periods of the pandemic, people were encouraged to work from home if possible, and upper secondary schools, universities, and other adult education institutions transitioned to distance learning. People over 70 were advised to stay at home and avoid going out to shop for food.

### Participants and data collection

Inclusion criteria were having participated in WeWorC, having experience in home care service work, and being currently employed in home care service. During spring 2022, and among the 154 interested people, we strived to include home care workers with a variation in terms of different geographical areas, workplaces, gender, and time of work experience to obtain different perspectives on the studied phenomenon. A total of fourteen staff members participated and were divided into five focus groups. The participants represented a variation in gender, workplaces, municipalities, counties (named A, B and C), in both rural and urban areas. Their median age was 52 years (range: 30–64 years). A compilation of demographic variation among the participants is found in Table 1.

**Table 1:** A compilation of the demographic variation of focus group participants

Interviews	Number of Participants	Men/Women	Mean age	Region A, B, or C
Focus group 1	4	1/3	53,8	2 from A, 2 from B
Focus group 2	3	1/2	47	1 from A, 1 from B, 1 from C
Focus group 3	3	1/2	38,7	1 from A, 1 from B, 1 from C
Focus group 4	2	1/1	47	2 from C
Focus group 5	4	2/2	51,8	2 from A, 1 from B, 1 from C

The participants represented a variation in gender, workplaces, municipalities, counties, in both rural and urban areas

Focus group interviews were adapted to prerequisites and working schedules to minimize the inconvenience for participants. Participants were contacted by telephone or mail in autumn 2022 and invited to join focus group discussions. All focus group interviews were conducted via the digital platform Zoom as a means of facilitating participation in the study. The counties in the study have a geographically widespread population, with long distances and lengthy travel between communities, which might have hindered in-person participation.

### Focus group interviews

To encourage interaction and discussions among participants, a semi-structured interview guide was employed. It was developed to address experiences of work environment and health and consisted of twelve open-ended questions. In short, the questions were about the perceived and experienced changes, challenges, and the effects on the work environment and health among the staff that resulted from the COVID-19 pandemic, as well as the support staff needed and lessons applicable to the future, see Appendix 1. All focus group interviews were conducted by pairs of interviewers from the research team, with a total of four interviewers involved. The duration of the interviews varied between 66 and 100 min. At the beginning of every focus group interview, the interviewers introduced themselves and presented the aim of the study. Furthermore, staff were asked about their work title, and workplace and informed about the importance of confidentiality. The researchers stressed that the things that were discussed during the focus group interview stayed within the group so that all participants felt that they could freely discuss the things they felt were important. Each focus group interview ended by asking the participants whether they had something to add and whether researchers could contact them again if anything was unclear in the focus group interviews. Focus group interviews were audio recorded by authors and transcribed verbatim by a transcription agency for further analysis. To verify that the transcribed focus group interviews were reliable, two of the researchers listened to parts of the focus group interviews and compared them with the transcripts. The researchers assessed that the transcripts were of high quality. Sound and transcribed text files were transferred and stored on a safe file area/space at the university.

### Analyses

Thematic analysis was used, which is a suitable method for qualitative data collected via semi- structured questionnaires [27]. The data analysis was inductive in nature, that is, identified themes are closer to the data than a pre-selected theory with a rich description of the dataset. The themes have then been identified based on the semantic nature of the codes rather than latent, underlying ideas, assumptions or ideologies. Essentialist epistemology formed the basis for the analysis, i.e., an assumption of a reality in which the individual attempts to understand their lived experiences by developing subjective meanings of certain objects or events in their context, like Braun and Clark, who also describe that meanings can be articulating through language [27, 31].

The dataset consisted of all transcripts of the focus group interviews. All text in the transcripts was initially read by all researchers familiar with the data set. After that, transcripts were coded by writing notes in the margins. A code could consist of one to several sentences and summarize something meaningful in the text, and the same excerpt from the transcript could be used in different codes. Coding was manifest in character, focusing on the semantic, explicit meaning of the data rather than looking for something beyond what a participant had expressed (or reading ‘between the lines’).To ensure trustworthiness of our data analysis, three researchers (KB, MÖ and AP-S) were enrolled in the coding process, with one transcript coded by one researcher and thereafter also coded by the other two researchers for purposes of validation. All authors discussed the coding and codebook and agreed that they saw the same meanings in the text.

In our analysis, we aimed for coherence by striving to be thoughtful, deliberative, reflexive, and theoretically aware of thematic analysis [31]. All codes from all focus group interviews were then analyzed to explore shared patterns capturing something important that had a distinct meaning in relation to the research question, i.e., thematization, see Appendix 2. The thematization was inductive in approach and was carried out by all three researchers together, and during this process, themes were reviewed to fit with the data.

In the results section, we have included quotes to illustrate the theme we have identified. In quotes where the statements are marked with letters, a dialogue arose between different focus group participants.

### Ethics

The research was approved by the Swedish Ethical Review Authority (Dnr 2021–02058). Participants received oral and written information about the study’s aim. The participants were informed that they could stop the focus group interview if they wished. They were also informed about the recording of the focus group interview, how sound and transcribed text files would be stored, and that transcripts would be handled with care so that no one could be identified in the text files. A written informed consent was given by all participants.

## Results

The overarching theme “expanded and unclear responsibilities characterized a pressured work environment during the pandemic” was chiseled out during analyses of the four sub-themes, see Table 2. The respondents stated that the COVID-19 pandemic significantly impacted the perceived work environment and health of home care service staff. The first subtheme summarized narratives about various aspects that jeopardized staff's own health. The second subtheme emphasized changes of work tasks and routines during the pandemic, with an emphasis on increased responsibility. The third subtheme delves into staffs’ experience of struggling between being a lifeline and being contagious to older adults, acting as a lifeline for them during isolation. Finally, the fourth subtheme emphasized the organizational challenges faced by staff, including the gap between leaders’ and staff members’ levels of competence.

**Table 2:** Overview of the thematic structure of data

Overarching theme	Expanded and unclear responsibilities characterized a pressured work environment during the pandemic			
Subthemes	My own health was jeopardized	A wind of change towards more responsibility	Struggling between being a lifeline and being contagious	Organization and management as facilitator or hindrance

### My own health was jeopardized

This subtheme encapsulates narratives concerning various aspects of well-being, such as personal health, stress, and the burden of a heavy workload. Participants experienced extremely low staff availability and high staff turnover due to sick leave and/or colleagues who quit during the pandemic and that was experienced as negative, as illustrated in the following quotation.

*“There has been extremely high staff turnover, which is never positive. Instead, it increases both stress and workload. I mean, there has been much, much higher absenteeism due to the directives that have been in place, requiring people to stay home at the slightest symptom. And that has necessitated more substitutes who haven’t been available…” (Focus group interview.5).*

“I suffer because I sweat and it's uncomfortable to wear a face mask, for example” (Focus group interview 4).

The high turnover was described as increasing workloads, which participants mentioned could result in tiredness and stress for staff on duty. Inconsistent staff availability led to the recruitment of staff through emergency solutions, potentially involving staff with low competence and a lack of training. Participants who had been healthy during the pandemic had to work overtime and introduce new staff. Uneven staff availability was noted to result in a loss of competence during certain periods within the home care team. The workload increased as existing staff members took on more responsibilities by overseeing additional advanced and delegated tasks that untrained staff could not handle. Participants mentioned that their own health was jeopardized and negatively affected due to a sense of moral stress that consisted of feelings of not being enough for the older individuals who needed their care.

*"we were told to treat it like a common cold……we were not supposed to assign more staff……and that created extreme stress for us, because, as I said, we work with people, this is a service profession. And, uh, we can’t, we don’t have the heart to leave a person who is so sick… uh… in their home. I mean, many live alone in houses, especially up there in (northern town), where I come from. Uh… It just doesn’t work that way” (Focus group interview 3).*

Participants spoke about their own health and wished that it had included a wellness break every week during the pandemic, like taking a walk or going for an extra coffee with a colleague.

### A wind of change towards more responsibility

This subtheme encapsulates narratives concerning changes in work tasks, work content, and routines that induced more responsibility for the elderly. Participants described how work methods and routines shifted during the pandemic, as exemplified in the following quotations where the participants state a responsibility for not transmitting the virus.

*“Yes, we received completely new equipment that we were not used to using…using protective gear, stuff like that…It became a completely different way of working, in that sense” (Focus group interview 5).*

*“And this distance that we must keep, uh, all the time, has been a big challenge […] you can't sit too close, you… can't communicate with, uh, hugs, and, uh… physical contact, which you're used to. Uh. Because a person might not, uh, remember your face when you come in. Or, if they have good hearing, they'll hear your voice, they'll feel the kindness in your voice. Uh. And then they'll recognize you when you come in and do what you always do, that you… You touch their shoulders, or that you put your hand on their back and pat their back when you talk, and… Because everyone has their own way, uh… when they're with a patient. And, when it (body language) suddenly… changes, then […] disappears, a small part of the security that they are… used to” (Focus group interview 2).*

Certain work methods, such as serving as a mentor giving support for the older adults, were emphasized as becoming even more important during the pandemic. The significance of providing support and assistance was heightened and strengthened.

*“And then during the entire pandemic… there were an incredible number of people who were, of course, cut off from the entire community. The users we go to. And we were the only ones they met” (Focus group interview 5).*

It was exemplified during focus group interviews that staff developed their skills during the pandemic and had to change their working methods to work more independently with more responsibility, making their own decisions.

“So, uh… no. Uh, we feel a little bit vulnerable here, without a manager, and… it gets a little… We must manage among ourselves” (Focus group interview 2).

The content of the days varied to a higher extent than had been the case before the pandemic. Hygiene routines were something that already existed, but staff described that they shifted during the pandemic to be more accurate and diligent in following said routines. They mentioned variations in hygiene practices among the staff, noting that some had exhibited a lazy attitude before the pandemic.

*“But that's the thing about… well, hygiene routines. That it… it's something that we've always done, but that… well, that we're constantly reminded of and… well, and we remind each other and… So that there's nothing really new about hygiene routines, but… But it became extra in focus, of course” (Focus group interview 5).*

Work methods changed during the pandemic with varying success. This increased responsibility was also evident in their narratives about protecting the older adult and compensating for what substitutes could not provide, which management had not taken responsibility for, such as replacing activities like walks. These types of activities were canceled without any replacement offered indoors. Cohort teams were created on the initiative of the healthcare organization, which facilitated coordination between staff and increased collaboration across geographical borders. This meant that certain staff could dedicate themselves solely to the older adults diagnosed with COVID-19.

*“And, I mean, then… when something like this happens, maybe, uh, you get a, uh,… a, user who has COVID-19. […] And then we would be there the whole time. […] then, there was a little, what do you call it? Argument among, among the staff. Yes, but, “why didn’t you come (to user X)?”, [..] “Yes, I’ve been (to users with Covid).” (Focus group interview 4).*

### Struggling between being a lifeline and being contagious

This subtheme encapsulates narratives concerning being indispensable and at the same time fearing transmitting infection. In some cases, during the pandemic, the participants were the only ones who interacted with the older adults; they spoke about having replaced loved ones without being able to provide that important sense of closeness. ‘Being a lifeline’ was also highlighted in participants’ description of their fear of transmitting infection to the elder individuals in their care and of being infected themselves. Being indispensable also involved a contradiction between wanting to care for the older adult but fearing bringing infection home to a vulnerable loved one. Some staff stopped seeing their family and friends out of fear of being infected; both for their own sake and to avoid transmitting the infection to the older adults.

Protective equipment proved to be cumbersome, particularly in cases where the older adult being cared for was affected by dementia, who sometimes interpreted the staff as thieves.

"I had a resident in my area who believed that there were robbers. (laughs) And that they would break into her home” (Focus group interview 2).

Conversely, when interacting with individuals who do not have cognitive impairments, there was a clear understanding of the importance of using protective equipment. However, for those with cognitive impairments, this understanding was lacking. Despite this, staff members in this scenario still implemented changes in their work routines, including the use of protective gear, driven by a sense of responsibility, loyalty to the organization, and confidence in the infection control unit.

*“[The older adult] prefer to see the person they are talking to. It's difficult when you have a face mask, actually. Working with visors and face masks is especially challenging for patients with dementia. They can't hear, they can't see, they can't perceive facial expressions. It has been a significant challenge, um"(Focus group interview 3).*

*“The most important thing is to, not to spread the infection [laughter]. You have taken care of your users and yourself and… if I may say so, to… well, to grow into this… role even more, to… protect and to be protected” (Focus group interview 4).*

Narratives related staff who were indispensable and created opportunities that contributed to older adults being able to stay in their homes. Using protective equipment could be perceived as hindering the relationship with individuals with poor hearing. Participants felt indispensable, and at the same time contagious. Many dementia patients are suspicious, and if someone comes in wearing protective equipment, they were often not perceived as particularly trustworthy. Even neighbors sometimes changed elevators when staff arrived, reinforcing the feeling of being seen as contagious. Being indispensable for older adults is exemplified in this quotation.

*"Throughout the entire pandemic, there were so many (older adults) who were isolated from the entire society…we were the only ones they met…it required so much more from us to keep perhaps the mood up, um, psychologically, um…What should I say? Motivate them that there will be a change…we became incredible mentors to our clients. More than we usually have been. Because we were the only ones…who stepped inside the door for many, many, many, many months” (Focus group interview 5).*

### Organization and management as facilitator or hindrance

Participants described that the organizations had a lack of flexibility, both before and during the pandemic, with interventions being highly detailed and controlled. This was perceived in the form of stricter controls that gradually increased over time, which were strenuous. During the pandemic, detailed control increased further, and the time allocated to each older adult was constrained or was not increased even with more work tasks to perform.

*“For that system, we have had it for as long as I have worked here,[…] There has been this standard time, and, […] not so much our boss, uh, but, uh… more our resource planner to whom we, uh, forward the requests, saying,"yes, we now need forty-five minutes instead of thirty-five,"or"now we need an extra half hour,"or"ten extra minutes there,"and so on, […]” (Focus group interview 2).*

“R; No, neither as S says, in the number of visit minutes that should be with that person or… no difference at all. Same race [laugh]. Or what do you say, S

S: Yes, no, exactly. No difference at all. No” (Focus group interview 5).

The narratives revealed that many individuals, including leaders, made significant efforts. Leaders who were described as attentive, organized, and clear were particularly appreciated. A resurgence of a mentality emphasizing the importance of both giving and taking was observed. The focus group interviews demonstrated a significant variation in descriptions of the organization and management. Several participants expressed strong loyalty to their managers and sometimes worked double shifts to support the organization and the older adults.

*“Because, we have changed a few managers over the years here, and, uh… But the, the, not the one we have now, but the one we had before, she would often send more, like,"Wow, you’ve been so good this weekend!","Oh, you can handle this!", and"what…". I mean, she really pushed you. I thought that was great” (Focus group interview 3).*

“Yes, of course, periodically there have been extra shifts, over time, and such. It was difficult to get hold of people, especially towards the end with the Omicron variant” (Focus group interview 5).

They also mentioned that in some organizations, there was no minute-by-minute control; instead, schedules were used as guidelines because, for example, a shower might take more time than the allocated duration. For staff, it is known when the older adults wish to have breakfast, want to shower, and take their medication, so planning is done accordingly, and assistance is offered if needed.

*“But, besides the manager setting the… shifts, we help each other, so… She doesn’t interfere, like, during the day, like that. […] Instead, we talk to each other. Yes. And then, we have, we talk often” (Focus group interview 4*).

Staff who described detailed control of their work mentioned that those in charge seemed unaware of what home care workers did during the workday. In some groups, it was highlighted that there was a lack of dialogue about how much time was needed for each individual user. This detailed control hindered flexibility, created time constraints, hindered well-being, and reduced desire to continue working within the organization.

“So the person who came up with… this thing with, the one who manages the standard times… They should have a… real understanding of, um… how much time is needed” (Focus group interview 2).

Staff had various ideas and proposals for work methods. In one focus group interview, it emerged that their own ideas received little attention from management. They expressed a wish that management and the organization would listen more closely to input from staff with extensive work experience and knowledge. For instance, a safety representative wanted to alter hygiene routines even before the pandemic but felt that the manager did not listen. Staff described that they had pointed out that the staff rooms were too small during the first wave of the COVID-19 pandemic and wondered what would happen in the case of new infections when they needed to maintain physical distance, but this had not been taken into consideration. Staff had wished to communicate COVID-19 results on individual occasions with the older adult, but results were exclusively communicated to the older adults by doctors or registered nurses, as illustrated in the following quotation.

*"The routine was that doctors were supposed to call and inform (about confirmed cases among residents)… And I can say that it [the organization] hasn't worked. We were not allowed to disclose it, so neighbors came and went, and there sat a positive case. Some have external cleaning companies, and there have been issues…” (Focus group interview 5).*

This was described as conflicting and a lacking routine, delaying the information, with the consequence that neighbors, relatives, and external services visited the older adult ignorant of the fact that they were infected, contributing to the spread of the virus.

## Discussion

In this study our aim was to illuminate experiences of the work environment and health challenges faced by home care workers supporting older adults during the COVID-19 pandemic. The main findings showed that an expanded and unclear responsibility characterized the work environment during the COVID-19 pandemic. Participants in the present study described that their health was jeopardized during the pandemic. They also narrated that they experienced changes in their daily work, with a focus on more responsibility, like becoming more indispensable to the older adults under their care. They described an increased workload due to several circumstances and were aware of organizational and managerial challenges. Our main findings will be discussed in relation to current research and subsequently synthesized against the JD-R model [20, 21, 32]. JD-R is a suitable model because it is context-based, i.e., it is intended to be used by all types of organizations because it does not specify which factors constitute demands or resources in the work. It all depends on the unique situation that employees and managers find themselves in [20, 32].

In the present study, participants highlighted that their own health was jeopardized due to an increased workload, as existing staff members shouldered additional advanced and delegated tasks that untrained and temporary staff could not handle. Furthermore, high staff turnover and increased levels of sick leave were also noted as escalating the workload, potentially leading to fatigue and stress for on-duty staff. The narratives predominantly revolved around mental burdens rather than physical tasks, which is interesting but explainable. A newly published systematic review synthesized staff’s experiences of mental burdens including depression and anxiety, which increased during the COVID-19 pandemic [33]. They concluded that there was a need for managers to prevent negative emotions among staff during a pandemic. In 2023, the Swedish Agency for Work Environment Expertise also reported that assistant nurses and registered nurses perceived higher emotional demands at work than quantitative demands [34]. This finding highlights the psychological strain faced by healthcare professionals in their roles. Furthermore, the same report showed that Swedish assistant nurses and nurses working in the municipal sector had turnover intentions as high as 49% and 42%, respectively. These turnover rates underscore the challenges faced by healthcare workers. Thus, when applying the JD-R model [20], the narratives in present study describe a health impairment process during the COVID-19 pandemic that in the end might affect the staff’s performance negatively. Further, high job demands can predict turnover intent [35].

A recent cross-sectional study which monitored home care nurses'heart rate and collected self-reported data on health, job demands, and work ability, concluded that home care work is classified as light-intensity based on their measured physical capacity in relation to physical work. Physical strain was reported to be higher among individuals aged 45 years and over, and during evening shifts compared to morning shifts [36]. A Swedish study confirms that a high workload affected older staff, and staff with more work experience more negatively than younger staff [13]. An interpretation is that the narratives in the present study predominantly revolved around mental burdens rather than physical ones. Therefore, prioritizing the development of knowledge to alleviate the mental burden of home care workers overall and physical burden of the older staff should be a focus in future research. In a report from the Swedish Agency for Work Environment Expertise (2023:13) there are evidence on health-promoting factors such as conditions that enable managers to lead and support their employees, stable work teams that can collaborate over time, the utilization of workplace competence and the importance of community and social support as significant health-promoting factor.

In the present study, results showed that work methods and routines shifted during the pandemic as a wind of change to more responsibility. Certain work methods, like mentoring the older adults, gained increased importance, and senior staff with more work experience took on more responsibility for supporting the older adults; i.e., by being a mentor. An interpretation is that there is a need to address the health of more senior staff to ensure high competence among staff and ensure the retention and recruitment of staff in the home care service. Nevertheless, the results of a systematic review revealed that, despite the assistance of technology in facilitating communication with relatives, many older adults remained dissatisfied. This dissatisfaction stemmed from older adults’ desire to visually connect with the entire person and to have a human touch in their interactions with relatives [33].

This feeling of being indispensable to older adults that was visible in the narratives implied being a crucial support, shouldering increased responsibility, being a substitute for family and relatives, and keeping older adults from being infected with COVID-19. Narratives highlighted staff's indispensability as the sole interaction for some older adults during the pandemic. Findings confirm that home care services staff experienced a professional pride and a duty to become a bridge between older adults and their relatives [33]. In addition, our results showed that home care service staff could experience potential contagion and faced suspicion from dementia patients when wearing protective equipment and suspicion from the older adults’ neighbors when visiting the individuals under their care. In contrast, Zhang et al., [33] reported that staff experienced fear of being infected, which could result in a reduction of the frequency of helping older adults with personal care. In a Japanese study, the results indicate that individuals receiving home care during the COVID-19 pandemic reduced their visits, and deprioritized social support and daily services such as cleaning [37] indicating a fear of receiving visits from home care staff.

This paradoxical situation—feeling that one is both indispensable and at the same time afraid of infecting frail older adults with COVID-19—can be called a ‘wicked problem’. A wicked problem, in short, is a decision or planning problem characterized by complex relationships with interdependent factors that affect each other in an intricate way, where different stakeholders see different solutions [38]. For example, the relatives of the older adults may emphasize the importance of hygiene routines to avoid infection and death and the older adults themselves may yearn for human touch. That might leave staff with a sense of “damned if you do, damned if you don’t”—a frustration that might result in stress and ill-health. Elsert Gynning et al., [34] found that both Swedish nurses and assistant nurses scored somewhat worse than the reference group on moral stress. In relation to JD-R (2014, 2017), moral stress is a heavy job demand.

The sense of being indispensable might act as a resource [39] as the researchers found that an orientation towards patient engagement seemed to reduce job demands and increased resources. Other researchers have found that the organization must work with occupational health interventions to create a good work environment for their healthcare staff [40]. In the present study, participants noted that the organization and management could be a facilitator or a hindrance. In line with JD-R [20, 32], facilitators at the workplace include resources that motivate staff and enhance performance. One of the facilitators was the formation of cohort teams to handle COVID-19 cases, which implied increased collaboration across geographical and/or organizational borders so that dedicated staff could focus only on older adults with COVID-19. A qualitative Swedish study confirm our results and described cohort-19 team care as a way of limiting the spread of COVID-19 [41, 42]. As this is an organizational issue—i.e., nothing that the staff themselves can decide upon—the organization must step in and create these cohort teams.

Another facilitator according to our study results was managers who were perceived as ready to listen, organized, and clear. Wallo and Lundqvist [43] found in their review that leadership is important for staffs'health and well-being through behaviors like inspiring, being a role model, stimulating, and motivating, and at the same time supporting and seeing each employee (i.e., transformative leadership). Health care organizations will find that transformational leadership is essential for maintaining and retaining nursing staff, as well as achieving overall patient satisfaction. It is imperative to focus on and evaluate the interactions between nursing managers and their staff. [44]. If leaderships show those behaviors, it qualifies as a resource, according to JD-R [20, 32].

When it comes to hindrances, our study results showed that the perceived degree of organizational control and time management was intensified during the pandemic in some municipalities. This prohibited flexibility in care and limited allocated time for each older adult. The narratives described a sense of management’s lack of awareness regarding staff members’ activities throughout the workday, and the unpredictable character of home care. Wallo and Lundqvist [43] show that when management demonstrates trust in the staff, gives them space and mandate and at the same time is flexible and adjusts its leadership behavior depending on staff’s needs and current situation, it benefits the staff’s work environment and health. Furthermore, the narratives contain information about staff proposing ideas for improvement but feeling their input was overlooked by management, leading to issues like delayed communication of positive COVID-19 results to their patients. This feeling of not being supported from management has also been reported elsewhere for assistant nurses through a lower rating of social support from managers than the reference value [34]. On the other hand, assistant nurses scored higher on social cohesion at work, indicating the importance of the working group.

However, home care service staff described a strong sense of loyalty and gave care regardless of unforeseen circumstances; sometimes working double shifts during the pandemic, a mentality of giving and taking. This mentality buffered organizational incapacity when it came to giving good quality care during the pandemic. A Swedish cross-sectional survey showed a similar finding in that there is an intertwined complexity of individual and organizational factors that affect the level of job strain among home care service staff. They suggested an implementation of new multidimensional work strategies that aim to reduce the level of job strain and thereby create a positive psychosocial work environment [45]. They also reported that home care service staff received the highest mean score in the item I want to do much more for older persons than my employers will allow, which is in line with participants’ descriptions in our study.

One interpretation suggests that strategic planning of shifts, considering staff members’ physical capacity, age, and incorporating time for recovery and social support in the work schedule could contribute to better health and job satisfaction for staff aged45 years and over. In contrast to younger counterparts, older staff often have more experience and, consequently, more responsibilities. The study by Sjöberg et al., [13] suggests that increased time to work on social support might strengthen staff in handling high workloads and thereby increase their health-related quality of life. Zhang et al. [33] imply that managers should formulate timely and effective management strategies to support staff during a pandemic. One interpretation is that fostering social support and open communication about organizational and individual factors that strengthen job satisfaction could also contribute to enhanced job satisfaction.

One dilemma that was described in the narratives was the organization surrounding delaying positive COVID-19 results to the older adults, which in some municipalities only could be handled by a doctors or registered nurses. This was described as delaying important information, with the consequence that neighbors, relatives, and external services visited the older adult without knowing that they were infected, contributing to the spread of the virus and in the end possibly infecting another frail older adult.

Furthermore, research has shown that moral stress is associated with low quality of care [46]. In the narratives, moral stress is often mentioned, and the participants see the relationship between moral stress, their own health, and quality of care. If management lacks the ability to pick up signals from their staff, which can be due to a number of factors, such as too high a workload on the part of the managers or a reluctance consider staffs’ suggestions, this will affect staffs’ motivation at work, which is in line with the JD-R model [20, 32].

An interpretation is that organizations should be prepared for the restructuring of healthcare that can and should be implemented during a crisis. For example, older adults with little need of daily care and significant local contacts and relatives may be prioritized as receiving a reduced amount of home care service, while older adults in great need of home care services may receive an increased number of visits and additional support in social aspects, especially when relatives cannot visit during a pandemic. Being prepared for the necessary priorities during a pandemic can alleviate the workload and mental stress placed on staff.

### Methodological considerations

The strengths of present study include the collection of data from a diverse group of staff members representing different geographical areas, ages, and work experiences, in line with [28]. All participants had worked in home care service during the COVID-19 pandemic, providing rich content in focus group interviews that deepened our understanding of their experiences and emotions. Conducting focus group interviews in pairs of interviewers facilitated natural dynamics and a rich flow of information, generating new insights and perspectives. Focus groups were beneficial due to the limited timeframe, allowing information gathering from numerous participants [29, 47]. Limitations include data collection from three northern counties in Sweden, which may affect transferability. However, we still expect results to be representative elsewhere in Sweden and to other countries with similar care organizations. Ensuring participants engage in dialogue aligned with the study's purpose was challenging, but having two interviewers provided some control.

Digital platforms like Zoom offered advantages, such as reaching geographically distant respondents, increasing inclusiveness despite shift work constraints [48]. Zoom interviews may lack the quality of face-to-face interactions, losing emotional information from body language, pacing, turn-taking, and silence. Respondents may not feel the same psychological security, affecting their willingness to share experiences. However, Zoom interviews have been safe [48] and more personal than email interviews [49]. In summary, Zoom focus group interviews were preferable for data collection. Moderators managed disadvantages by distributing the floor, clarifying rules, and addressing misunderstandings.

To achieve trustworthiness, the study ensured findings were credible, transferable, dependable, and confirmable [50]. Senior interviewers were familiar with nursing, home care, work environment challenges, and organizational psychology. Background knowledge shaped positions, focusing on work environment and health experiences of employees. Triangulating results through co-coding and repeated discussions minimized researcher biases. Careful study design, prior knowledge, and data analysis aimed for transferability, allowing replication by qualitative researchers. Dependability was maintained through detailed notes on progress and decision-making. Researchers considered their influence on the process, building on a study from 2017.

## Conclusions

This study shed light on how home care service staff experienced their work environment and health during the COVID-19 pandemic. The main findings highlighted expanded and unclear responsibilities that characterize the pressured work environment, which can lead to increased health risks for participants. Work methods and routines shifted during the pandemic, with a notable increase in responsibility, and participants faced challenges in implementing protective measures.

The health risks associated with increased workload, stress, and mental burdens were evident in the narratives. Existing research corroborates these findings, emphasizing the need for management strategies to support staff mental health during routine work and during pandemics. The Job Demands-Resources (JD-R) model is applicable here, illustrating how organizational aspects can act as both demands and resources.

Suggestions for future research include exploring strategies to enhance job satisfaction, such as fostering social support and open communication about organizational factors. The implications of a changing climate, which might imply new pandemics, that co-varies with a major demographic shift are very real. For employers, this means preparing for future crises such as pandemics and striving for a good work environment with tolerable conditions so that the home care service can recruit staff and retain those who already exist. It is also of great importance that different authorities in healthcare and social care create effective cooperation so that information, knowledge and policies are easily disseminated to frontline staff who have the concrete responsibility for our elderly.

## Supplementary Information

Supplementary Material 1.

Supplementary Material 2.

## Data Availability

The data that support the findings of this study are not openly available due to reasons of sensitivity and are available from the corresponding author upon reasonable request. Data are located in controlled access data storage at Umeå University.
