# Supplementary material for: Expanded and unclear responsibilities: the evolving role of home care workers as a lifeline during the COVID-19 pandemic -a focus group interview study
Source: BMC Health Serv Res. 2025 Aug 22;25:1120. doi: 10.1186/s12913-025-13145-2 (PMC12372233; doi:10.1186/s12913-025-13145-2)
Supplement: Supplementary file 1 — Supplementary Material 1. [file 12913_2025_13145_MOESM1_ESM.docx]

### Interview Guide for Focus Group Interviews with Home Care Workers

The focus group interview is estimated to last about 90 minutes.

**Information about:**

- Brief introduction about the Project
- Everyone introduces themselves
- Confidentiality and Non-Disclosure Agreement
- Audio Recording and Data Storage Information
- Consent to Participate in the Study
- Any Questions or Concerns before the Interview start?
- Audio Test Recording

**Interview questions:**

*Organization*

- Have the organization and work methods changed during the pandemic? If yes, please describe how?
- What challenges have you experienced during the pandemic?
- Which situations have been particularly challenging?
- I there anything else you´d like to share about transitioning from regular care work to working as care during a pandemic?
- What aspects of the organization have promoted your work environment during the pandemic?
- Have these changes impacted your health? If so, please explain in what ways?
- What could help promote your health during the pandemic?
- What specific support do you need to function effectively as care staff during a pandemic?

*Staff Engagement*

- What motivates you in your role as care staff?
- Would you describe an ideal role model for home care personnel?
- What lessons from your experience will you carry into the future as a home care personnel?
- What expectations or wishes do you have for your employer in order to continue working as home care personnel in the future?
- What lessons from the pandemic could improve home care services?
- How would you like to develop your workplace to improve the work environment for yourselves!

*Concluding Questions*

- Is there anything you´d like to share about home care service work in general, or specifically during the pandemic, that we haven’t covered?
- Do you see yourself working in home care service in the next five years?

*Follow-up Questions during the Interview Adapted to the Conversation*

- Can you tell me more about…
- Can you give examples of…
- Do you mean that…
- Would you like to add anything?
