# Supplementary material for: Expanded and unclear responsibilities: the evolving role of home care workers as a lifeline during the COVID-19 pandemic -a focus group interview study
Source: BMC Health Serv Res. 2025 Aug 22;25:1120. doi: 10.1186/s12913-025-13145-2 (PMC12372233; doi:10.1186/s12913-025-13145-2)
Supplement: Supplementary file 2 — Supplementary Material 2. [file 12913_2025_13145_MOESM2_ESM.docx]

**Appendix 2**

### Table 1. Examples of thematization

| Excerpts from transcripts | Codes | Themes | Overall Theme |
| --- | --- | --- | --- |
| There has been extremely high staff turnover, and that is never positive. Instead, it increases both stress and workload, Interview 5. | Extremely high staff turnover increases stress and workload | My own health was jeopardized | Expanded and unclear responsibilities characterized a pressured work environment during the pandemic |
| It’s terrible. I mean, after vacuum clean an apartment, just wearing a mask… I mean, that mask is used up. It only takes ten minutes, (laughs) and you’re already sweaty, interview 4. | Terrible wearing mask, only few minutes becoming sweaty |  |  |
| There have been several changes in routines…  Regardless of the test result, we use protective equipment until we receive the outcome. | Several changes in routines | A wind of change towards more responsibility |  |
| There have been several changes in routines. If someone exhibits cold symptoms, we must contact the district nurse. They will automatically conduct a COVID test. | We had to contact the district nurse |  |  |
| We are like… Sometimes like mom and dad, sometimes like siblings, sometimes like a brother or a sister, or something like that. Many cases when, when you have been to someone enough, you almost become like family at their home. | We became their family | Struggling between being a lifeline and being contagious |  |
| We work with people, this is a service profession. And, uh, we can't, we don't have the heart to leave a person who is so sick... uh... in their home. Well, they, many people live alone at home | Many elderly were so alone |  |  |
| And that, when we then get these routines. There have also been some things that have been less good. Eh, our municipal management has sent them out, on, on the intranet, and you might think my immediate boss will come out with it as soon as she, eh, nah, but then it is referred to reading routines. […] . It's tough, reading a routine. It's easier to read it and have it described to you verbally. Eh, "this is how it is, now we're going to go out with this, now we're going to work this way". That, and there are assumptions, and... | Would have liked to have both written and verbal information about new Covid routines. | Organization and management as facilitator or hindrance |  |
| [...] uh, the communications manager, for the entire municipality. [...] and, and, well, as you say, when there's a lack of... uh, uh... protective equipment. Then he went, down to, uh, I don't know if it was (large city) or (medium-sized city) and got it. | Senior managers took matters into their own hands and solved problems |  |  |
